# Basic education teachers post-COVID-19 pandemic: prevalence of disease symptoms and association with health habits and behaviors

**DOI:** 10.47626/1679-4435-2024-1258

**Published:** 2025-01-07

**Authors:** Thalita Cardoso Souza, Claudiana Donato Bauman, Rose Elizabeth Cabral Barbosa, Rosângela Ramos Veloso Silva, Desirée Sant’Ana Haikal, Nayra Suze Souza-e-Silva

**Affiliations:** 1 Department of Physical Education and Sports, Universidade Estadual de Montes Claros (Unimontes), Montes Claros, MG, Brazil; 2 Graduate Program in Health Sciences, Unimontes, Montes Claros, MG, Brazil; 3 Graduate Program in Primary Health Care, Unimontes, Montes Claros, MG, Brazil

**Keywords:** occupational health, school teachers, education, primary, and secondary, COVID-19, health surveys, saúde ocupacional, professores escolares, ensino fundamental e médio, covid-19, inquéritos epidemiológicos

## Abstract

**Introduction:**

COVID-19 is an infectious disease, caused by the SARS-CoV-2 virus, which may be associated with health problems arising after recovery from disease-specific symptoms; these are understood as sequelae.

**Objectives:**

To analyze health problems arising from COVID-19 and their associated factors among basic education teachers in the state of Minas Gerais, Brazil.

**Methods:**

Epidemiological, cross-sectional, analytical study, using an online survey design, carried out with teachers working in basic education in the public school system. Data collection took place between October and December 2021 using an online form. The dependent variable was health problems resulting from COVID-19, categorized as no vs. yes. Descriptive bivariate and multiple analyzes were carried out using Poisson regression.

**Results:**

A total of 1,907 teachers participated in the study, 77.2% of whom were female, 94.4% were under 60 years old, 21.4% were part of at least one high-risk group for COVID-19, and 99.5% had been vaccinated against COVID-19. Of the total number of teachers, 19.7% were diagnosed with COVID-19; among these, 41.9% reported some health problem resulting from COVID-19. There was a higher prevalence of health problems resulting from COVID-19 among teachers who reported poor sleep quality (prevalence ratio = 1.79), who were depressed (prevalence ratio = 1.39), and who were part of at least one high-risk group for COVID-19 (prevalence ratio = 1.46)

**Conclusions:**

Among teachers who contracted COVID-19, a substantial prevalence of health problems resulting from this infection was observed, as well as major health outcomes associated with these problems.

## Abstract (pt)

**Introdução:**

A covid-19 é uma doença infecciosa provocada pelo vírus SARS-CoV-2, podendo ocasionar problemas de saúde após a recuperação dos sintomas específicos da doença, sendo eles entendidos como sequelas.

**Objetivos:**

Analisar os problemas de saúde decorrentes da covid-19 e seus fatores associados entre professores da educação básica do estado de Minas Gerais.

**Métodos:**

Estudo epidemiológico, transversal e analítico, do tipo *websurvey*, realizado com professores atuantes em escolas da educação básica pública. A coleta de dados ocorreu entre outubro e dezembro de 2021 por meio de formulário on-line. A variável dependente, categorizada em não *versus* sim, foram os problemas de saúde decorrentes da covid-19. Foram realizadas análises descritiva, bivariada e múltipla, utilizando a regressão de Poisson.

**Resultados:**

Participaram do estudo 1.907 professores, dos quais 77,2% eram do sexo feminino, 94,4% tinham menos de 60 anos, 21,4% faziam parte de pelo menos um grupo de risco para covid-19 e 99,5% se vacinaram contra a doença. Do total de professores, 19,7% foram diagnosticados com covid-19, e, entre eles, 41,9% relataram algum problema de saúde decorrente da infecção. Houve maior prevalência de problemas de saúde decorrentes da covid-19 entre os professores que relataram qualidade de sono ruim (razão de prevalência [RP] = 1,79), que estavam com depressão (RP = 1,39) e que faziam parte de pelo menos um grupo de risco para covid-19 (RP = 1,46)

**Conclusões:**

Foi observada uma prevalência expressiva de professores que foram infectados pela covid-19, que sofreram de problemas de saúde decorrentes da infecção, bem como de importantes desfechos de saúde associados a esses problemas.

## INTRODUCTION

COVID-19 is an acute infectious respiratory disease caused by the SARS-CoV-2 virus.^1^ The disease spread rapidly worldwide due to its high transmissibility and was ultimately classified as a pandemic. The coronaviruses are a broad group of viruses that infect several animal species and can cause mild respiratory illness, with the potential to progress to severe disease.^2^

Many people recover from COVID-19 and continue to experience symptoms beyond the expected period, which are understood to be sequelae of the infection. These symptoms include cardiorespiratory fatigue, sleep problems, memory loss, psychological disorders, muscle pain, and headaches, among others.^3-5^

During the COVID-19 pandemic, schoolteachers faced a host of financial, social, and cultural problems due to major changes in learning and teaching practices.^6^ This was the greatest challenge ever faced by the education system, which led to disruption of conventional education and a transition from face-to-face, in-person teaching to remote learning activities.^7^ Considering the extent to which the lifestyle habits of teachers and students have changed during the pandemic period, mental health problems such as anxiety, loneliness, and depression have all been aggravated by this unexpected process.^8^

Work is not only a source of remuneration and self-actualization of various human needs, but it also provides a means for maintenance of interpersonal relationships and survival. However, it can also be a source of illness when it poses health hazards or risk factors and workers lack strategies to protect themselves from these hazards.^9^

For teachers in particular, the devaluation of public education, rising violence in the classroom, and a lack of student discipline have been considered triggers for various health problems (both physical and mental), resulting in dissatisfaction with work and even illness.^10^ Within this context, the present study aimed to analyze health problems resulting from COVID-19 and their associated factors among basic education teachers in the state of Minas Gerais (MG), Brazil.

## METHODS

### STUDY DESIGN

This epidemiological, cross-sectional, analytical study was conducted using an online survey design. The study followed the recommendations of the Checklist for Reporting Results of Internet E-Surveys (CHERRIES)^11^ and the Strengthening the Reporting of Observational Studies in Epidemiology (STROBE)^12^ guidelines.

### PARTICIPANTS

The study was carried out with a population of approximately 90,000 teachers working in basic education in the MG state-school system (data provided by the MG State Department of Education [SEE/MG]), distributed across approximately 3,500 schools. The state of MG is made up of 853 municipalities, with a population of 20,539,989, according to the 2022 Census.^13^

### SAMPLE SIZE AND CONTEXT

To calculate the sample size, a formula based on the prevalence of the disease or event of interest was used, considering an infinite population. A prevalence of 50% was considered, with an error rate of 3% and a 20% increase to make up for potential losses. We therefore estimated that data had to be collected from 1,282 teachers to ensure the representativeness of the sample for the state of MG.

Initially, authorizations were obtained and a partnership with SEE/MG was established. Once authorized, data collection took place from 26 October through 31 December 2021, by means of an online questionnaire made available to teachers via the Google Forms platform. The link to the data collection form was disseminated by SEE/MG, an invitation was published on its official website (<https://www.educacao.mg.gov.br>) and emails were sent to the 45 Regional Education Superintendencies (*Superintendências Regionais de Ensino*, SREs) across the state, with each SRE being asked to forward the invitation to its teachers’ institutional email addresses. The link to the data collection form was sent at two time points: on the first day of data collection and approximately 30 days later.

The data collection form was preceded by an Informed Consent Form (ICF) regarding participation in the study. Teachers were also asked directly whether they agreed to take part in the study and were offered the possibility of printing out an ICF signed by the study coordinator if they so desired. According to data from a pilot study carried out with 16 teachers from different municipalities across MG, completion of the data collection form took approximately 40 minutes. To avoid automatic form-filling by robots, an image-based CAPTCHA challenge-response system (Google reCAPTCHA) was used.

Each teacher’s email address and unique public servant identifier with check digit (MASP) were collected to ensure that the study sample was reaching its intended audience only and prevent duplicate filling of the questionnaire. All data tabulation, systematization, and analysis procedures were carried out using only the codes assigned to each returned form.

The study included teachers who were teaching in the year of data collection, taught in primary and/or secondary schools, were employed by the MG state school system, and agreed to take part in the study. The exclusion criteria were teachers who were working in non-teaching functions (e.g., school principals), retired teachers, and those who did not agree to participate in the study.

### INSTRUMENTS AND VARIABLES

The dependent variable of this study was health problems resulting from COVID-19. This variable was formulated on the basis of two questions: “Were you diagnosed with COVID-19 at any time during the pandemic?”; for respondents who answered “yes”, a follow-up question was asked: “Have you had any health problems resulting from COVID-19?”. Both were dichotomous yes-or-no questions.

The following independent variables were considered: sex (female vs. male), age (under 60 years vs. 60 years or older), quality of life (good vs. poor), sleep quality (good vs. poor), anxiety (no vs. yes), depression (no vs. yes), diet (healthy; needs changes; inadequate), physical activity (yes vs. no), excess weight (no vs. yes), high-risk group for COVID-19 (no vs. yes) and vaccinated against COVID-19 (no vs. yes).

Sleep quality was assessed with the following question: “Over the past month, how would you rate the quality of your sleep overall?” For the anxiety and depression variables, teachers were asked whether they had received a medical diagnosis of these mental health problems, considering the last 12 months preceding data collection.

Dietary assessment was performed with a validated, 24-item instrument which aims to measure healthy eating practices. The questions cover four dimensions: Planning, Domestic organization, Modes of eating, and Food choices. All items are multiple-choice, scored on a four-point Likert-type scale. The total score is obtained by the sum of the items (1 to 24) and is categorized as follows: unhealthy diet (up to 31 points), changes needed (between 31 and 41 points), and healthy diet (above 41 points).^14^

Physical activity level was assessed with the International Physical Activity Questionnaire (IPAQ), short form, as validated for Brazil.^15^ In accordance with World Health Organization (WHO) guidelines,^16^ teachers who engaged in at least 150 minutes of physical activity per week were classified as active, while those who engaged in less than 150 minutes or no physical activity at all were classified as inactive.

Weight status was calculated using the teachers’ self-reported weight and height and classified according to the cutoff points established by WHO^17^ as normal weight (body mass index ≤ 24.9 kg/m^2^) or overweight (body mass index > 25 kg/m^2^). To ensure greater reliability of results, teachers who reported being pregnant at the time of data collection were excluded from the analysis of weight status.

### STATISTICAL ANALYSIS

Data were analyzed in SPSS Version 22.0. The frequency and prevalence of the variables were described. Bivariate analysis was performed using Pearson’s chi-square test to observe the relationship between health problems resulting from COVID-19 and the independent variables. Only variables with a p-value ≤ 0.20 on bivariate analysis were initially selected to compose the multivariate model, conducted using Poisson regression with robust error variance, with the category of interest being teachers who reported health problems resulting from COVID-19.

A process of backward elimination was used, i.e., the variables with p ≤ 0.20 were entered all at once into the model and removed one by one considering the p-value and the goodness of fit. Only variables with a descriptive level < 5% (p < 0.05) were retained for the final model. The magnitude of the associations was estimated by crude and adjusted prevalence ratios (PRs), with 95% confidence intervals (CIs) and a significance level of 5% (α ≤ 0.05).

### ETHICAL ASPECTS

The ProfSMinas project was authorized by SEE/MG and approved by the Research Ethics Committee of Universidade Estadual de Montes Claros (Unimontes), with opinion no. 4,964,125/2021. Teachers were informed of the methodology of the study, its objectives, and the confidentiality of the information, and an ICF was made available. The study was also conducted in accordance with Brazilian National Health Council Resolution 466/2012, which sets forth rules for research involving human subjects.

## RESULTS

A total of 1,907 teachers from approximately 354 municipalities across the state of MG participated in the study; 10.6% taught in rural areas. Among the overall sample, 77.2% of respondents were female, 94.4% were under age 60 years, 39.2% reported poor sleep quality, 38.6% were anxious, 21.4% were part of at least one high-risk group for COVID-19, and 99.5% had been vaccinated against COVID-19. These and other data are given in Table 1.

**Table 1:** Sample profile: basic education teachers in the state of Minas Gerais, 2021 (n = 1,907)

Variable	n (%)
Sex	
Female	1,473 (77.2)
Male	434 (22.8)
Age^*^ (years)	
Less than 60	1,800 (94.4)
60 or older	106 (5.6)
Quality of life	
Good	1,804 (94.6)
Poor	103 (5.4)
Sleep quality	
Good	1,160 (60.8)
Poor	747 (39.2)
Anxiety	
No	1,171 (61.4)
Yes	736 (38.6)
Depression	
No	1,658 (86.9)
Yes	249 (13.1)
Diet	
Healthy	971 (50.9)
Changes needed	738 (38.7)
Unhealthy	198 (10.4)
Physically active^*^	
Yes	625 (35.0)
No	1,162 (65.0)
Overweight^*^	
No	645 (34.7)
Yes	1,212 (65.3)
High risk for COVID-19	
No	1,498 (78.6)
Yes	409 (21.4)
Vaccinated against COVID-19	
Yes	1,897 (99.5)
No	10 (0.5)

* n is variable due to missing data.

Of all teachers included in the study, 19.7% (n = 373) had been diagnosed with COVID-19. Among these, 41.9% reported some health problem resulting from the infection.

Various health problems resulting from COVID-19 were reported by teachers, such as memory loss (25.3%), persistent fatigue or malaise (24.5%), body aches (21.3%), shortness of breath (9.9%), gastrointestinal problems (7.7%), skin infections (5.9%), and cardiovascular disorders (5.3%).

Table 2 shows the association between reported health problems resulting from COVID-19 and variables related to health habits/behaviors. On multivariable analysis, the prevalence of health problems resulting from COVID-19 was higher among teachers who also reported poor sleep quality (PR = 1.79), who were depressed (PR = 1.39), and who were part of at least one high-risk group for COVID-19 (PR = 1.46) (Table 3).

**Table 2:** Bivariate analysis of independent variables in relation to health problems resulting from COVID-19 among basic education teachers in the state of Minas Gerais, 2021 (n = 375)

Variable	Health problems resulting from COVID-19		p-value
	No n (%)	Yes n (%)	
Sex			0.145
Female	167 (56.2)	130 (43.8)	
Male	51 (65.4)	27 (34.6)	
Age^*^ (years)			0.379
Less than 60	207 (57.7)	152 (42.3)	
60 or older	11 (68.8)	5 (31.3)	
Quality of life			0.004
Good	208 (60.3)	137 (39.7)	
Poor	10 (33.3)	20 (66.7)	
Sleep quality			< 0.001
Good	153 (70.2)	65 (29.8)	
Poor	65 (41.4)	92 (58.6)	
Anxiety			< 0.001
No	144 (68.6)	66 (31.4)	
Yes	74 (44.8)	91 (55.2)	
Depression			< 0.001
No	201 (62.4)	121 (37.6)	
Yes	17 (32.1)	36 (67.9)	
Diet			0.024
Healthy	105 (63.6)	60 (36.4)	
Changes needed	94 (57.3)	70 (42.7)	
Unhealthy	19 (41.3)	27 (58.7)	
Physically active^*^			0.003
Yes	79 (69.3)	35 (30.7)	
No	125 (52.7)	112 (47.3)	
Overweight^*^			0.291
No	71 (62.3)	43 (37.7)	
Yes	141 (56.4)	109 (43.6)	
High risk for COVID-19			0.003
No	188 (61.8)	116 (38.2)	
Yes	30 (42.3)	41 (57.7)	
Vaccinated against COVID-19			0.492
Yes	215 (58.0)	156 (42.0)	
No	3 (75.0)	1 (25.0)	

* n is variable due to missing data.

**Table 3:** Health problems resulting from COVID-19 among basic education teachers, state of Minas Gerais, 2021 (n = 375)

Variable	PR (95%CI)	p-value
Sleep quality		< 0.001
Good	1.00	
Poor	1.79 (1.39-2.30)	
Depression		0.008
No	1.00	
Yes	1.39 (1.09-1.77)	
High risk for COVID-19		0.001
No	1.00	
Yes	1.46 (1.16-1.85)	

PR = prevalence ratio.

## DISCUSSION

This study sought to identify health problems resulting from the COVID-19 pandemic among public school teachers working in basic education in the Brazilian state of Minas Gerais. Among the teachers investigated, 19.7% had been diagnosed with COVID-19; of these, 41.9% reported at least one health problem resulting from infection with the novel coronavirus. As reported in the literature, patients may experience several debilitating symptoms or signs during or after COVID-19 infection.^4^

Among the teachers evaluated in the present study, health problems resulting from the COVID-19 pandemic included memory loss, shortness of breath, persistent fatigue or malaise, sleep problems, and body aches. These findings are in line with national and international studies, which have also identified multiple symptoms, diseases, or health problems as post-COVID conditions.^18,19^ However, for teachers, the illness-wellness continuum is also constructed in the workplace, their work being of a biological, psychological, and social nature. One’s work environment can be a space for reaffirming self-esteem, developing new skills, and expressing one’s emotions, but can also expose one to occupational diseases, jeopardizing physical and mental health.^20^

The present study found that 58.6% of teachers who reported health problems resulting from COVID-19 also had sleep problems. The prevalence ratio of 1.79 indicates that teachers with health problems related to COVID-19 infection are 79% more likely to have poor sleep when compared to teachers who did not develop health problems as a result of COVID-19 infection. Prolonged sleep deprivation can have significant adverse effects on mental and physical health. Sleep problems also impact overall quality of life and are considered a risk factor for future absenteeism and functional disability among workers.^21,22^

Considering the Brazilian adult population, Barros et al.^19^ found that those who did not have sleep problems before the pandemic began to experience such problems, while those who already had a history of sleep problems saw their conditions worsen.

The present study also found an association between having some health problem resulting from COVID-19 infection and a diagnosis of depression. The mental health problems and stress already experienced by teachers were compounded by the pandemic, which required all teaching staff to adapt quickly to provide access to a variety of educational materials remotely and virtually, as well as work to prevent the spread of disease in the classroom. Faced with this situation, many teachers reported high levels of stress, burnout, anxiety, and depression.^23^

The present study validates the findings of Perez-Cano et al.,^3^ who identified emotional markers (such as anxiety, depression, and stress) resulting from COVID-19 in almost half of the population investigated. COVID-19 has had a negative impact on the global education system, particularly when school closure mandates were implemented. The onset of the pandemic forced the whole world to implement emergency measures, which included suspending in-person classes.^24^

From this perspective, the presence of mental disorders, psychological distress, and poor sleep quality has negative impacts on health and quality of life, contributing to a significant percentage of years lived with disabilities. Mental distress can trigger risk factors for chronic noncommunicable diseases and viral infection, and during pandemics, the incidence and severity of emotional disorders and insomnia tend to rise.^19^

In addition to the aforementioned health problems, social distancing played an important role in safeguarding the physical integrity of the population, reducing the spread of the virus during the pandemic; however, the longer the time spent apart from others, the greater the risk of developing psychological issues such as depressed mood, irritability, anxiety, fear, and anger. Social distancing has also increased the adoption of unhealthy and high-risk behaviors, such as increased intake of alcoholic beverages, tobacco, and illegal substances.^25,26^

The findings of the present study show that teachers who reported being in at least one high-risk group for COVID-19 had a higher prevalence of health problems resulting from COVID-19 infection. The scientific literature has validated several such risk factors, including female sex, belonging to an ethnic minority, socioeconomic deprivation, and early dyspnea. The multifaceted nature of COVID-19 and its epidemiological complexity make several organ systems vulnerable to infection, including the nervous, cardiac, respiratory, vascular, cutaneous, and hematopoietic systems, as observed in the course of this investigation.^27-29^

Some limitations of the present study must be taken into account. Because of its online survey design, the study was subject to selection bias, as it depended on internet access for teachers to complete the instrument. Recall bias was also a concern, since responses were based on self-report. Nevertheless, such research designs also have benefits, such as the possibility of collecting data remotely, reaching a larger population group, achieving a broad geographic scope, streamlined planning, and rapid publication of results.^30^ Additionally, we consider the institutional support of SEE/MG in carrying out the research, especially at the time of data collection, as a strength of this study.

## CONCLUSIONS

The present study demonstrated a substantial prevalence of public-school teachers who contracted COVID-19 and went on to develop health problems as a result of this infection. Among the most prevalent problems were memory loss, persistent fatigue/malaise, and body aches and pains. Having health problems resulting from COVID-19 was also associated with other important health factors among teachers, such as sleep problems, a diagnosis of depression, and belonging to a high-risk group for COVID-19 infection.

These findings demonstrate how the pandemic was harmful to this population, leading to health problems beyond those expected to be caused by the disease itself. Furthermore, they reaffirm the need for further studies to identify and control new health outcomes arising as a result of COVID-19.
